# PINE NANOCELLULOSE AND BACTERIAL NANOCELLULOSE DRESSINGS ARE SIMILAR IN THE TREATMENT OF SECOND-DEGREE BURN? EXPERIMENTAL STUDY IN RATS

**DOI:** 10.1590/0102-672020200002e1533

**Published:** 2020-11-20

**Authors:** Guilherme Andrade COELHO, Maria Angélica Baron MAGALHÃES, Alysson MATIOSKI, Jurandir Marcondes RIBAS-FILHO, Washington Luiz Esteves MAGALHÃES, Francine Ceccon CLARO, Rafael Koerich RAMOS, Thayline Mylena Santana de CAMARGO, Osvaldo MALAFAIA

**Affiliations:** 1 Postgraduate Program in Principles of Surgery, Mackenzie Evangelical Faculty of Paraná/Medical Research Institute, Curitiba, PR, Brazil; 2 EMBRAPA Forests, Colombo, PR, Brazil

**Keywords:** Wound healing, Bandages, Therapeutics, Nanotechnology, Cicatrização, Bandagem, Terapêutica, Nanotecnologia

## Abstract

Background: Despite all the advances in medicine and the wide variety of dressings available, the treatment of burn wounds still represents an important medical challenge. The pinus cellulose membrane dressing is a biomaterial with characteristics similar to those of bacterial cellulose, but with lower cost.

**Aim:**

To evaluate the efficacy of pinus nanocellulose membrane on healing of deep second degree burns in rats and compare with Membracel®.

**Method:**

Thirty male Wistar rats were submitted to deep second degree burn in dorse, with boiling water at 97o C for 20 s, generating a 314 mm² area wound. The animals were distributed in three dressing groups (n=10): group 1 - simple gauze; group 2 - bacterial cellulose membrane (Membracel®); and group 3 - pinus cellulose membrane. They were evaluated for 20 days to verify clinical condition, macro and microscopic appearance and wound contraction.

**Results:**

All of them remained clinically well with no differences in weight. Crusts were observed in group 1, and none in groups 2 and 3. Regarding to scar contraction, groups 2 and 3 were similar, better than group 1. Microscopic analysis showed predominance of advanced healing degree in groups 1 and 3, and initial in group 2. Mature collagen was predominant in all groups.

**Conclusion:**

The pinus nanocellulose membrane is effective in the treatment of experimental second degree burn in rats and its effectiveness is similar to that of the bacterial nanocellular membrane.

## Graphical Abstract

[Fig f1a]

**Figure f1a:**
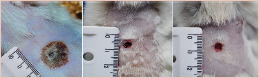
Crusts in 7^th^ follow-up day: G1 yes; G2 no; G3 no (left to right)

## RESUMO

**Racional:**

Apesar de todos os avanços da medicina e da grande variedade de curativos disponíveis, o tratamento das queimaduras ainda representa importante desafio médico. O curativo de membrana de celulose de pinus é biomaterial com características semelhantes à de celulose bacteriana, mas de menor custo.

**Objetivo:**

Avaliar a eficácia da membrana de nanocelulose de pinus na cicatrização de queimaduras profundas de segundo grau em ratos e comparar com a Membracel®.

**Método:**

Trinta ratos Wistar machos foram submetidos à queimadura profunda de segundo grau em dorso, com água fervente a 97o C por 20 s, gerando lesão de 314 mm². Os animais foram distribuídos em três grupos de curativos (n = 10): grupo 1 - gaze simples; grupo 2 - membrana de celulose bacteriana (Membracel®); e grupo 3 - membrana de celulose de pinus. Eles foram avaliados por 20 dias para verificar o quadro clínico, aspecto macro e microscópico e a contração da ferida.

**Resultados:**

Todos permaneceram clinicamente bem, sem diferenças de peso. Crostas foram observadas no grupo 1 e nenhuma nos grupos 2 e 3. Em relação à contração da cicatriz, os grupos 2 e 3 foram semelhantes, melhores que o grupo 1. A análise microscópica mostrou predomínio de grau de cicatrização avançado nos grupos 1 e 3, e inicial no grupo 2. O colágeno maduro foi predominante em todos os grupos.

**Conclusão:**

A membrana de nanocelulose de pinus é eficaz no tratamento de queimaduras experimentais de segundo grau em ratos e sua eficácia é semelhante à da membrana nanocelular bacteriana.

## Graphical Abstract

[Fig f1as]

**Figure f1as:** Crostas no 7º PO: G1 sim; G2 não; G3 não (esquerda para direita)

## INTRODUCTION

Despite the large number of techniques and products available for the treatment of burn wounds, failures are still widespread. Among the barriers to treatment, the difficulty in closing the skin is of great importance, for it brings death of cels, risk of infections, and other comorbidities, prolonging hospital stay and encumbering treatment. As a result, it is necessary to look for inexpensive and effective products to optimize the healing process of burn patients.

There are lots of different wound dressing avaiable, but none of them fullfils the needed requirement^15^ and, despite all the advances, the use of these resources is not always accompanied by success. The high cost of available products makes treatment more expensive and, sometimes, impractical. Cellulose nanofibers has gained special attention in recent years due to their large availability, biocompatibility and physical-chemical characteristics, which gave them numerous biomedical and pharmaceutical applications^9^. 

Bacterial nanocellulose membrane (BNM) has been used since the 1980s. Its main properties are pain and inflammation control, maintenance of moisture, growth and defense factors, favoring granulation and reepithelization. Besides, it allows a lower contamination rate, visualization of the exudate appearance and less frequent dressing changes^21^.

One established option in the market is Membracel^®^ (Vuelo Pharma, Curitiba/PR, Brasil), a bacterial cellulose dressing that temporally replaces the skin. It is a biocompatible, inert, and resistant dressing that allows the reduction of healing time. Besides, the diary dressing change is not necessary, optimizing its use and reducing the treatment costs^24^. 

The good results obtained with BNM stimulated this research with pine nanocellulose. 

Given the circumstances surrounding the treatment of burn wounds and possible failures with existing techniques, there is a need to create a simple, effective, and available dressing for these injuries. In this context, the production of a vegetable cellulose dressing from agribusiness products, with similar characteristics to those of bacterial cellulose and Membracel^®^, could reduce costs and favor sustainability, besides creating new options for the treatment of burn patients. 

This research aimed to verify the clinical safety and the effect of pinus nanocellulose membrane (PNM) in the healing process of deep second-degree burn in rats and to compare it to the commercial BNM (Membracel^®^) safety and effectiveness.

## METHODS

This study was carried out on the premises of the Experimental Surgery Laboratory of the Institute of Medical Research at Makenzie Evangelical Faculty of Paraná, Curitiba, PR, Brazil. The work was conducted in accordance with the Technical Standards for Animal Research and Experimentation and with Law 11,794, of October 8, 2008. It was submitted to the approval of the Ethics Committee for the Use of Animals by FEMPAR.

### Production of cellulose nanofibrils and the membranes

The cellulose nanofibrils from pine (*Pinus taeda*) were produced at Wood Technology Laboratory in Embrapa Florestas (Colombo, PR, Brazil).

The nanocellulose membranes were produced from a cellulose nanofibrils suspension in pure water - a gel. This gel of nanocellulose resulted from the defibrillation of bleached pulp cellulose, which were done by the mechanical super grinding technique using a lab equipment Supermasscolloider mill (Masuko JP).

**FIGURE 1:**
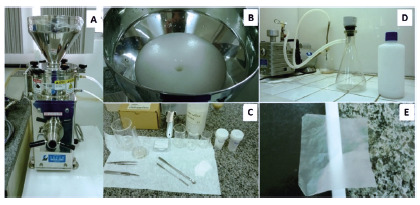
Experimental procedure for pine cellulose nanofibrils production: A) Supermasscolloider grinder from Masuko JP; B) bleached cellulose pulp being processed by the mill; C) typical glassware used to produce nanofibrillated cellulose; D) vacuum filtration for manufacturing of nanocellulose membranes; E) fabricated nanocellulose membrane

Briefly, 2% of the dry cellulose pulp bleached in pure water is first homogenized in a laboratory blender to obtain a fluffy paste. This suspension is then passed 30 times through the colloidal supermass crusher, adjusting the distance between the silicon carbide crushers.

The nanocellulose gel is vacuum filtered in a polyamide membrane filter to prevent adhesion of the formed cellulose membrane and is easily removed after drying. During drying at 60o C, a 6 mm thick flat glass was used to press the cellulose membranes and keep them free from wrinkles between two polyamide filters. The amount of 2% nanocellulose gel was calculated to result in a nanocellulose membrane with a nominal weight of 30 gm-2 (Figure 1). Before applyed to the animal´s wounds, the PNM were sterilized in ethylene oxide. 

### Group distribuction

We studied 30 male adult Wistar rats (*Rattus Norvegicus albinus*, rodentia mamalia). At baseline, all animals were 120 days and weighed 150 g. They were distributed randomly in three groups (n=10): group 1 (control - G1) - deep second degree burn in the skin of dorsus and second intention healing; group 2 (G2) - deep second degree burn in the skin of dorsus and treatment with BNM (Membracel^®^); group 3 (G3) - deep second degree burn in the skin of dorsus and treatment with PNM.

### Anesthesia

The rats were anesthetized with intramuscular 2% xylazine hydrochloride (Xilazin^®^, Syntec do Brasil, Cotia, SP, Brail), at a dose of 10 mg/kg, associated with 10% ketamine hydrochloride (Cetamin^®^, Syntec do Brasil, Cotia, SP, Brazil), at a dose of 60 mg/kg. For previous analgesia, an intramuscular dose of morfin (Morfina^®^, Cristalia, SP, Brazil, 0,5 mg/kg) was applied 10 min before procedures. 

### Burn induction

In all animals, deep second-degree burns were induced in dorsal skin, with 20 mm diameter and 314 mm² of area. The measurement of wound area was obtained from the formula of the circular area, in wich: circle area =π x radius^2^ and once: π=3.14 and wound radius=1 cm (10 mm) 

**FIGURE 2:**
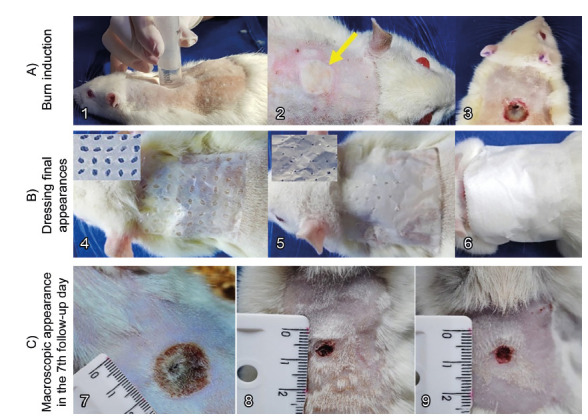
A) *Burn wound induction*: 1) boiling water inside 20 ml syringe; 2) final aspect of the burn injury - observe the pale skin area surrrounded by a hyperemic halo (arrow); 3) appearance of the wound three days after the burn induction - observe the scab formation. B) *Dressing final appearances*: 4) group 2 dressing made with BNM (Membracel^®^); 5) group 3 dressing made with PNM; 6) simple gauze and crepe bandages. C) *Macroscopic appearance in the 7* ^*th*^ *follow-up day*: 7) group 1, completely covered by crusts; 8) reddish wound with no crusts in group 2; 9) same, in a rat of group 3.

The wound was the same in all the rats and followed a template made with a 20 ml syringe that had its distal end cut across to generate a circular orifice to delimit the area of the injury. The device was pressed on the animal’s back, then filled with 10 ml of boiling water (at 97° C), with the aid of another syringe (Figure 2A1). The hot water remained for 20 s in the rat skin and a stopwatch was used to control this time. Subsequently, the device was removed, taking care that water does not fall into the rat’s body, performing a uniform wound (Figure 2A2). The water temperature was monitored with a thermometer. 

Imediatly after performing the burn, we removed a small skin fragment (1 mm^2^), includind subcutaneous tissue, to confirm the burn deph. The tissue sections prepared for histology were stained with H&E. Then, the injury was left open in the animals from three groups to observe the evolution and crust formation. 

During the first three days after burn induction, there was a crust formation over the lesion in each animal (Figure 2A3). On the third day, the crust was removed by debridement, creating an open wound. Then, the nanocellulose dressing (BNM and PNM) was put over de burn in the animals from groups 2 and 3, respectively. In group 1, was performed a simple gauzing dressing. 

In group 1, wounds were treated with simple gauze dressing soaked in 0,9% saline solution and then covered with crepe bandages (Figure 2B6). In group 2, the BNM dressing (Membracel^®^) was applied over the lesion (Figure 2B4) leaving about 0,5 cm leftover the size of the wound and then covered with dry gauze pads. In group 3, the procedures were the same as group 2, but the wounds covered with PNM dressing (Figure 2B5). The trunk of all the animals of the three groups was bandaged with crepe bandages to prevent contamination of wounds and the removal of dressings by the rats. The membranes were replaced every seven days or always when removed by the rats. 

After procedures, antibiotic prophylaxis was performed by subcutaneous injection of amoxicillin 20 mg/kg every 24 h for five days. Analgesia was achieved by subcutaneous administration of tramadol at dose of 5 mg/kg every 12 h for five days.

The animals were followed daily for 20 days to observe systemic and local parameters. To detect toxicity, was verified clinical parameters such as general state (movement, alertness and responsiveness to the environment), appetite, mortality and weight variation during follow-up period. 

To detect allergic reaction, inflammation, infection, or other local complications, the wounds were verified always when the dressings were remade. The macroscopic examination consisted of verifying the presence of exudate, bleeding, phlogistic signs (hyperemia, heat, pain and edema), abscesses and necrotic tissue (scabs). Was also verified if the membranes and dressings withdrawal caused damage to the wounds, discomfort, or pain to the rats.

### Follow-up

At the end of the follow-up period, the rats were weighted and sacrified under anesthesia with ketamine (200 mg/kg). Were removed two skin fragments from the edge of the scar for histological analysis.

The wound area was measured using digital pachymeter DC-60^®^ to obtain the horizontal and vertical measurements of each wound. The measurement of all wounds was always performed by the same researcher. In addition, another measurement was taken by another researcher, without knowledge about the evaluated group. Then, was calculated the percentage of contraction of each lesion using the mathematical model proposed by Agren et al.^1^, in which the percentage of contraction (CP) consists of the result of the final area (AF) minus the initial area (AI), divided by the initial area and multiplied by 100.

$$PC\;\left(\%\right)\;=\;\left[FA\;–\;IA\;\left(mm^{2}\right)\;/\;IA\;\left(mm^{2}\right)\right]\;X\;100$$

### Microscopic analyses

The tissue sections prepared for histology were stained with hematoxylin and eosin (H&E) and picrosirius red (PR). The H&E stained sections were examined under optical microscope Axiovision software (Carl Zeiss^®^), and their digital images were analyzed with Axiovision software (Carl Zeiss^®^). The PR stained sections were examined under optical microscope with polarized light to determine the concentration of type I and type III collagen fibers. These digital images were analyzed with Image-Pro Plus 4.5^®^ program, which recognizes thick and red fibers (mature type I collagen), and green and thin collagen fibers (immature type III).

The microscopic study consisted of verifying the healing score (1, 2, 3 and 4), and the percentage of type I and type III collagen.

The healing scoring relied on the degree of cellular invasion, granulation tissue formation, fibroblasts, vascularity, and re-epithelialization^8^. Score 1 consisted of wounds with minimal cell accumulation, lack of granulation tissue or epithelialization. Sections classified as score 2 had thin and immature granulation tissue filled by inflammatory cells, but with few fibroblasts, capillaries or collagen deposition, and minimal epithelial migration. The score 3 included wounds with moderately thick granulation tissue, filled predominantly by fibroblasts and extensive neovascularization. Collagen deposition was moderate, and epithelium can range from minimal to moderate migration. Score 4 consisted of abundant granulation tissue and neovascularization, filled by fibroblasts and extensive collagen deposition; wounds were covered by partial to complete epithelialization, with or without crust. 

### Statistical analysis

The data were presented as mean ± standard error of the mean. To compare continuous variables in different groups, we verified the data normality using the Kolmogorov-Smirnov test, followed by analysis of variance (ANOVA) and multiple post-hoc comparison LSD test (least significant difference) the Kruskal-Wallis test for nonparametric data. To compare the final weight and weight variation, we used the covariance analysis model (ANCOVA) adjusted for the initial weight. Categorical variables were described by frequency and percentage and compared using the Chi-square test and the adjustment of logistic regression model exact test. All results were considered significant for a probability of significance greater than or equal to 95% (p≤ 0.05).

## RESULTS

### Clinical evaluation

Anesthetic induction, as well as the performance of burns, evolved without major complications. All animals remmained well, could drink water and eat food without difficulty throughout the experiment.

The replacement of wound dressings did not cause damage to the regenerated wounds or pain to the animals, as the membranes could be easily detached from the wound surface. Stripping off gauzes, however, resulted in slight damage and bleeding to the wounds, and discomfort to the rats.

### Weight gain

There was no difference between groups as to animal´s weight before procedures and on the last day of follow-up (p=0,410, Table 1). 

**TABLE 1:** Values (mean±standard error of the mean) of the weight and weight variation of the animals in groups 1, 2 and 3, obtained before burn induction and in the last follow-up day.

Group	Weight (in grams)		
	Day 0	Day 20	Weight variation
1	129.0±12.6	197.3±25.2	68.3±18.8
2	143.2±8.4	203.5±9.6	60.3±9.0
3	131.0±14.5	196.7±20.3	65.7±13.3

1=control group; 2=group 2; 3=group 3; day 0=before burn induction; day 20= last day of follow-up

### Macroscopic appearance of the wounds

There was no bleeding, local inflammation or abscess in the wounds of groups 2 and 3. In group 1, the wounds were covered by scabs, even after debridement (Figure 2C7). In the animals of groups 2 and 3, the wounds remained reddish and covered by a thin layer of neoformed epithelial tissue (Figures 2C8 and 2C9).

### Scar contraction

When comparing the final area, absolute scar contraction and percentage of scar contraction, was found difference between groups (p<0,001). Comparing the values between the groups, was found difference between groups 1 and 2 (p< 0,001), as well as between groups 1 and 3 (p< 0,001); but, there was no difference between groups 2 and 3 (p=0,348), which means that scar contraction in groups 2 and 3 was similar and higher than in group 1 (Table 2).

**TABLE 2:** Values (mean ± standard error of the mean) of final area (FA, mm^2^), absolute car contraction (mm^2^) and percentage of scar contraction of the animals in groups 1, 2 and 3

Group	Final area (mm^2^)	Absolute scar contracion (FA - IA, mm^2^)	% Scar contraction (% of inicial area)
1	91.4±38.3	222.6±38.3	70.9±17.0
2	38.3±25.3	275.7±25.3	87.8±11.2
3	27.2±15.3	286.8±15.3	91.3±6.8

1=control group; 2=group 2; 3=group 3; FA=final area; IA= nicial area

### Microscopic analysis

#### Burn depth

**FIGURE 3:**
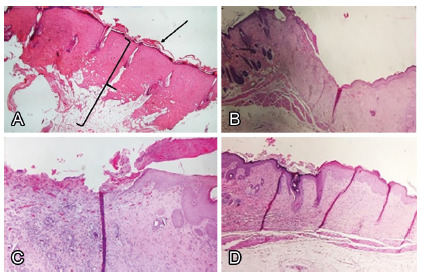
A) Microscopic aspect of the wound immediately after burn, showing complete injury of dermis and epidermis (note the preserved lamina basal -arrow -, as well as the muscle and adipose tissue - bracket); B) microscopic appearance showing the healing stage in the last follow-up day, group 1, with medium thickness granulation tissue, stage 3; C) group 2, filled with immature granulation tissue, inflammatory cells, capillaries and some fibroblasts, with lack of epithelium in most of the wound, stage 2; D) group 3, with thick and vascularized granulation tissue, recovered by epithelium, stage 4 (H&E x40)

In all of the animals, the burn reached the dermis completely, preserving the adjacent subcutaneous tissue, consisting of a deep second-degree burn (Figure 3A). 

#### Healing score

In the animals of groups 1 and 3, there was a predominance of wounds in an advanced stage of healing. In these groups, the wounds were partially or totally epithelized, filled with thick granulation tissue and abundant neovascularization (Figures 3B and 3D). In the animals of group 2, most of the wounds were in the initial stages of healing, filled with small and immature granulation tissue, inflammatory cells, capillaries and some fibroblasts (Figure 3C).

Comparing the frequency of healing scores, was found difference between groups (p=0.034). The values were different between groups 1 and 2 (p=0.025). However, they were similar between groups 1 and 3 (p=0.284) and between groups 2 and 3 (p=0.131, Table 3).

**TABLE 3:** Absolute and relative frequency of healing scores found in the animals of groups 1, 2 and 3, on the last follow-up day.

Group	Healing score			
	1	2	3	4
1	0 (0%)	1 (10%)	3 (30%)	6 (60%)
2	0 (0%)	7 (70%)	3 (30%)	1 (10%)
3	0 (0%)	3 (30%)	3 (30%)	4 (40%)

Groups: 1=control; group; 2=group 2; 3=group 3; score 1=lack of granulation tissue; score 2=thin and immature granulation tissue, minimal epithelialization; score 3= moderate granulation tissue, neovascularization and moderate epithelialization; score 4=abundant and vascularized granulation tissue, partial to complete epithelialization.

### Percentage of type I and type III collagen

The percentage of type I and type III collagen was similar between groups (p=0,449, Table 4). There was moderate to abundant collagenization, predominantly of mature collagen, characterized by thick fibers stained with red. 

**TABLE 4:** Percentage (mean±standard error of the mean) of type I and III collagen fibers in the wounds of the animals of groups 1, 2 and 3, on the last follow-up day

Group	Collagen I (%)	Collagen III (%)
1	80.9±20.7	19.1±20.7
2	78.1±16.0	21.9±16.0
3	72.8±17.9	27.2±17.9

Groups: 1=control group; 2=group 2; 3=group 3

**FIGURE 4:**
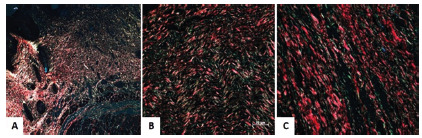
Photomicrograph of collagen fibers of the animals of groups 1. 2 and 3: A) predominance of mature collagen fibers. but desorganized and full of gaps in group 1 rats; B) mature. thick and organized collagen fibers in group 2; C) and also in group 3 (PR)

A smaller proportion of young collagens was observed in the three groups. characterized by fine fibers stained with green (Figure 4). Collagen was disorganized and full of gaps in the animals of group 1 (Figure 4A) but organized in groups 2 and 3 (Figures 4B and 4C). 

## DISCUSSION

Despite the medical and bioengineering advances treatment of burn wounds still represents a clinical and surgical challenge especially when they complicate. There are a vast number of available products. However, their results remain doubtful. 

Cellulose-based polymers are very abundant and available products with a high potential for biomedical and biotechnology applications. Cellulose nanofibers have several advantages to be used in the treatment of wounds, such as biocompatibility, maintenance of the moisture, resistance, and low toxicity ^6,13,14,17,20,22,23,25^. Besides the bacterial^17^ and viral growth^26^ could be reduced in the presence of the nanocellulose. These properties add excellent characteristics to the nanocellulose dressing^9^.

Bacterial nanocellulose membranes (BNM) have been used successfully in the treatment of burns and other wounds. Studies show improvement in the healing process and reduction of healing time with these wound dressings^3^ ^,^ ^12^ ^,^ ^19^ ^,^ ^21^ ^,^ ^24^. Membracel^®^ is a bacterial nanocellulose-based wound dressing very used in the treatment of burn wounds which could act as a temporary skin substitute. It is biocompatible, inert, atoxic, and has good resistance in the moist environment. The presence of pores allows gas exchange and the passage of exudate into a secondary dressing. Because of its characteristics the daily chances are not necessary avoiding damage to the wound bed and favoring the healing tissue. These properties give Membracel^®^ characteristics close to the ideal dressing^24^. However, its relatively high cost limits its high-scale use by the health system. 

Due to the good results obtained with BNM in the clinical routine we aimed to investigate the effect of PNM in the treatment of burn wounds. The vegetable cellulose is chemically and physically similar to bacterial cellulose, but with lower production cost which stimulated the development of this research to evaluate its effects on the healing process.

The present research is one of the first to evaluate the effect of vegetable cellulose nanofibers in the tissue healing process and is the first in the literature to report its use in the treatment of burn wounds. 

We chose the deep second-degree burn as it is the most frequent depth present in burn patients^2^. Additionally, more superficial burns heal spontaneously while the deepest ones usually require skin graft which is not the aim of this research. In the present study all lesions fully reached the dermis confirming the efficacy and effectiveness of the model proposed here to produce a deep second-degree burn. This data is relevant to standardize the burn lesions and consequently the results of the study which is of great importance for the study of burn wounds.

The assessment of behavior, weight gain, and early recovery in animals allows us to observe the systemic response due to tissue injury. The presence of pain and agitation in animals of group 1, and absence in groups 2 and 3, show the efficiency of the membrane in minimizing patient discomfort. Adequate pain relief has a positive effect on the speed and quality of recovery from tissue damage^9^. which corroborates the results of the present study. 

The macroscopic parameters here studied allowed us to verify the evolution of the wounds, signs of inflammation and infection, as well as the speed of healing. We did not observe any case of necrosis, intense inflammatory reaction, purulent exudate, or abscesses in both groups treated with cellulose dressings, similar to previous studies using bacterial nanocellulose^5^ ^,^ ^9^ ^,^ ^24^. These data suggest that PNM, as well as BNM, allows the drainage of the wound, without favoring the dryness of the covered area and bacterial contamination, two of the main factors that compromise healing. In the present study, we did not observe reactions of sensitivity to the vegetable membrane, such as edema, flushing, pain. or itching, confirming the clinical safety for its use in wounds. Other previous studies have also confirmed the biocompatibility of nanocellulose-based dressings, with no signs of allergic and inflammatory reactions resulting from their use^5^ ^,^ ^9^. These data confirmed positive results concerning biocompatibility and clinical safety and could ensure the absence of any toxic effect triggered by the PNM, as well as other nanocellulose-based wound dressing tested before^13^ ^,^ ^17^ ^,^ ^21^ ^,^ ^24^. 

To measure the wound area and scar contraction, we used a pachymeter, as Yaguishita (2007)^24^ and Agren et al. (1997)^1^. It allows us to verify the scar contraction ratio, which was higher in the animals of groups 2 and 3, corroborating with other studies^14^ ^,^ ^16^ ^,^ ^24^. Although there was no significant difference in wound closure between the two groups, the average scar contraction and final area in group 3 were higher than in group 2. These results suggest that PNM was more efficient in wound contraction than the other tested dressings.

We have no subsidies to say that the PNM has properties of tissue repair, otherwise, it may serve as a matrix for cells and proteins, which naturally participates in the healing process. The nanocellulose-based membrane could favour proliferation. cell migration and epithelialization from the edges to the center of the lesions. improving the wound healing^7^ ^,^ ^10^ ^,^ ^13^ ^,^ ^14^. 

In both cellulose dressing groups, there was no contamination of the wound site, and the moisture was maintained probably by the wound cover with porous dressing. Drainage of secretions through the pores hinders bacterial growth and keeps the environment moist. This factor results in an improvement of 35-45% in the rate of epithelialization of wounds^9^. Contamination of the wound is one of the most damaging factors to healing due to the release of cytotoxic enzymes by bacteria and by the exudation process itself, which creates a mechanical barrier to the action of the defense and healing cells. In the present study we have no subsidies to affirm that BNM avoided the bacterial growth, once we did not perform a cell culture. However, clinical and macroscopic valuation suggest they did not support the growth of pathogens. Nanocellulose-based wound dressing has the potential to impair bacterial biofilm growth. These tested membranes may cover the wounds. protecting it towards external microorganisms^9^. 

Membracel^®^ has pores created with position memory, which means that they do not change in diameter over time and allows the continuous exchange of fluids^24^. As a result, in the tested PNM, we performed pores manually to allow the drainage of secretions. As it is a new product in the initial testing phase and not yet commercialized there is no standardization of the size and shape of the pores. With the conclusion of this research, we will conduct further studies to develop a standardized dressing in thickness, size, and shape of the porous, to start the tests in clinical patients. 

Important characteristics for a wound dressing are the ability to absorb fluids and exudates, as well as easy removal from the wound, without causing pain or injury to the healing tissue^9^ ^,^ ^13^. In our study, the replacement of nanocellulose dressings (BNM and PNM) did not cause damage to the regenerated wounds, as the membranes could be easily detached from the wound bed, as observed by Hakkarainen et al. (2016)^9^. Stripping off gauzes, however, resulted in slight damage to the wounds, which prolonged the recovery. Gauze dressing has great permeability. but tight adhesion to the wound, causing pain and tissue injury when removed^18^. Furthermore, it supports the dryness of the wound, impairing cell migration, and consequently the healing process.

In all of the rats of group 1, crusts were present and each withdrawal and cleaning caused trauma, bleeding, and a painful reaction. Besides, the presence of necrotic tissue predisposes to bacterial growth and impair the action of the fibroblast^11^. Nanocellulose dressing promotes autolytic debridement, reduces pain, and accelerates granulation, which are crucial characteristics for wound healing^9^.

At day 20, a superficial neoepithelium layer covered the wound surface, and neovascularization was observed at the wound sites in group 3. We also observed an advanced healing stage in group 1, but the epithelialization was still scarce. However. most of the wounds at group 2 were in initial stages of healing, contrary to Yaguishita (2007)^24^ and Lin et al. (2013)^13^. These authors found an advanced stage of healing in wounds treated with BNM on the 20^th^ day. These divergent data could be explained by the different kinds of developed wounds, as they studied acute lesions, and we performed burn wounds. Tissue necrosis resulting from scald may have compromised healing, which may explain the divergence between the works. The results of the present research suggest that PNM support the healing process of burn wounds better than the other tested products. 

The PR stain allows us to verify the extent and concentration of collagen, as well as to differentiate mature and immature fibers. Healthy dermis has 80% of type I collagen and 20% of type II, while granulation tissue presents 30 to 40% of type III [34]. In our research, the percentage of collagen was similar between the groups, contrary to Yaguishita (2007)^24^ who found a higher proportion of mature collagen in animals treated with Membracel^®^. Probably. it occurred due to the local we removed the skin sections for analysis. Yaguishita removed the entire wound for histological study, being able to establish a more reliable assessment regarding the proportion of collagen and the differences between the groups. 

In the present study we removed the skin sections only on the edge of the wound, where the healing process starts. Thus, it tends to be similar between groups in the analyzed period. The mechanism of contraction is the centripetal movement of the wound margins to the center of the lesion. It plays a fundamental role in reducing the wound area, healing by secondary intention, as in our experiment. Reepithelialization occurs by the migration of epithelial cells from preserved attachments and structures of the dermis. Migration and proliferation begin during the proliferative phase and occurs until the complete closure of the wound and the membrane barrier re-established^11^.

Another difference between our research and Yaguishita´s work was the follow-up period^24^. This author observed the animals weekly, for 28 days, while we made just one microscopic analysis in the 20^th^ follow-up day. However, the time does not seem to be of importance for the different results, but rather the local of removed skin. Yaguishita did not observe difference in the percentage of collagen after 14 days. His data, added to our results, were consistent with those of previous studies. which says that the wounds have 80% of mature collagen (type I) on the 20^th^ day^11^.

Our microscopic findings (H&E and PR) showed that the initial healing process, which starts in the margins of the wound, is similar between the three groups. However, macroscopic examination showed the wounds treated with PNM and BNM healed more rapidly, as compared with those treated with simple gauze dressings. These results were consistent with those of several previous studies, showing that nanocellulose-based dressings support the healing process^4^ ^,^ ^7^ ^,^ ^9^ ^,^ ^10^ ^,^ ^13^ ^,^ ^14^.

The scar contraction of the animals treated with PNM, as well as the other macroscopic findings, did not differ from those treated with BNM. These data confirm the efficacy of the newly tested product for the treatment of second-degree burn. The shorter healing time added to the lower chance of bacterial growth, minimizes the length of hospital stay, as well as the need for systemic and topical medications, consequently reducing costs and clinical complications. Considering the vast number of burned patients seen daily around the world, it is of great importance to create an effective, less expensive, and affordable dressing.

In this research we did not evaluate the costs of the treatment with PNM, but preliminary studies suggest that it is significantly cheaper than other available products widely commercialized. 

The main benefit of wood nanocelluloses is its producibility from the inexhaustible cellulose source of plants, added to its lower production cost. Thus, it could be possible to create a cheaper and more accessible product for all classes. Besides, the shortest healing time and the low probability of infection represent important advantages for using the PNM in the treatment of burn wounds.

We have continued with further planned studies concerning the use of the PNM in the treatment of burn wounds, its effect on bacterial growth and deeper lesions, and its production costs. We aim to standardize the PNM dressing and extent its use for clinical trials. We do not intend to replace products available on the market. but to include PNM as an accessible resource for the treatment of burn wounds.

## CONCLUSION

The pine nanocellulose membrane dressing is safe and effective for the treatment of deep second-degree burn wounds in rats. Its results are similar to those from the commercial bacterial cellulose dressing (Membracel^®^). 
